# Full-endoscopic decompression for thoracic ossification of ligamentum flavum: surgical techniques and clinical outcomes: A retrospective clinical study

**DOI:** 10.1097/MD.0000000000022997

**Published:** 2020-10-30

**Authors:** Wenyi Li, Shangju Gao, Long Zhang, Can Cao, Jingchao Wei

**Affiliations:** Department of Orthopedics, Hebei General Hospital, Shijiazhuang, Hebei, P.R. China.

**Keywords:** dural ossification, minimally invasive, thoracic ossification of ligamentum flavum, thoracic spinal stenosis

## Abstract

**Background:**

Many complications are associated with thoracic open decompression surgery, such as dural tears and neurological deficits. The clinical outcomes are also not satisfactory. Full-endoscopic decompression of the lumbar spinal canal has achieved satisfactory results for the treatment of lumbar spinal stenosis. This surgery may be used for the treatment of thoracic ossification of the ligamentum flavum (OLF) under local anesthesia. The aim of our study is to introduce the surgical techniques used for full-endoscopic decompression for thoracic OLF and to evaluate its safety and efficacy.

**Methods:**

Fourteen patients with thoracic OLF (4 combined with dural ossification) underwent full-endoscopic decompression surgery. An interlaminar approach was performed. The anchoring method was used to establish the working passage. Spinal cord exposure began at a space between the ossification and the spinal cord, and dorsal and contralateral decompression were performed with the “Over the Top” technique. The modified Japanese Orthopedic Association score (11 points) was used to evaluate the efficacy during follow-up. At the same time, the visual analogue scale score for assessing back pain before and after the operation was evaluated.

**Results:**

The average operation time was 159.73 ± 62.09 minutes, and the hospitalization time was 7.43 ± 1.79 days. The follow-up period ranged from 8 to 22 months. Neurological function was improved. There were no serious complications. Dural tears occurred in 5 patients, intraoperative neurological deterioration occurred in 1 patient, and intraoperative headache and neck pain occurred in 1 patient.

**Conclusion:**

Full-endoscopic decompression is an effective, safe surgical technique for thoracic OLF even the cases combined with dural ossification.

## Abstract 2

Supplemental Digital Content is available in the text

## 1 Introduction

Thoracic ossification of the ligamentum flavum (OLF) is one of the causes of thoracic myelopathy, especially in Asian countries, such as China, Korea, and Japan.^[1,2]^ Thoracic OLF accounts for 50% to 60% of all patients with thoracic spinal stenosis, and the pathogenic factors are still unclear.^[3]^ Surgical treatment is widely accepted as the best treatment for thoracic OLF. The main surgical method is laminectomy combined with or without internal fixation, which can fully decompress the spinal cord after the operation. However, the clinical efficacy and the incidence of complications after surgery are often unsatisfactory.^[3]^ The mean complication rate is 24%. Cerebrospinal fluid leakage is the most reported postoperative complication (19%).^[4]^

With the development of surgical techniques and the updating of surgical equipment, full-endoscopic decompression of the lumbar spinal canal has achieved satisfactory results for the treatment of lumbar spinal stenosis. In particular, good outcomes and advantages of full-endoscopic decompression via an interlaminar approach have been described by many doctors for the treatment of lumbar spinal stenosis, which is characterized by hypertrophy of the ligamentum flavum and convergence of articular processes.^[5]^ The use of full-endoscopic thoracic spinal decompression for OLF has rarely been reported. In this study, 14 patients with thoracic OLF underwent full-endoscopic decompression surgery via an interlaminar approach in our hospital from March 2018 to December 2018, 4 of whom also had dural ossification (DO). During a follow-up of 6 to 18 months after surgery, neurological function and the visual analogue scale scores improved. There were no other complications except asymptomatic dural tears in 5 patients, intraoperative neurological deterioration in 1 patient, and intraoperative and postoperative headache and neck pain in 1 patient.

The objective of our study is to introduce the full-endoscopic decompression for thoracic OLF especially cases combined with DO and to evaluate the safety and efficacy. In this article, we report the key points of surgery and the clinical outcomes, especially the complications and remedies.

## 2 Materials & methods

### 2.1 Ethics approval and consent to participate

This study was approved by the ethics committee of our hospital and was performed according to the ethical standards outlined by the 1964 Declaration of Helsinki and its later amendments or comparable ethical standards. Informed consent was obtained from all individual participants included in our study.

### 2.2 Clinical data

From June 2017 to December 2018, 14 patients with thoracic myelopathy caused by thoracic OLF underwent this surgery in our department. The inclusion criteria were as follows:

1. patients with clinical manifestations of thoracic myelopathy, such as walking instability, numbness and/or weakness of the lower extremities, and muscular hypertonia;
2. the main pathogenic factor was OLF with single-segment or multi-segment spinal stenosis, which was clinically determined to be the main responsible segment; and 3. the clinical manifestation was consistent with imaging.

The exclusion criteria were as follows:

1. combined with high-grade deformity, instability, or prior surgery in the target segment, where a high-grade deformity was defined as a Cobb angle greater than 30 degrees^[6]^ and where instability was defined as associated soft tissue or osseous anomalies in the target segment^[7]^; and
2. fracture, infection, or tumor of the spine.

### 2.3 Statistical analysis

All statistical analyses were performed with SPSS software (version 23; IBM Corp., Armonk, NY) using paired *t* tests. The threshold for significance was *P* < .05.

## 3 Surgical methods

### 3.1 Surgical instruments

The internal diameter of the endoscope was 4.3 mm, and the working length was 181 mm. The view angle was 30°. A 2.5 mm diameter Kirschner wire was used to anchor the lamina. The surgical power device was equipped with 2 drill sizes: 3.5 mm and 3.2 mm. Other instruments were the same as those used for percutaneous endoscopic lumbar discectomy.

### 3.2 Operating method

**Figure 1:**
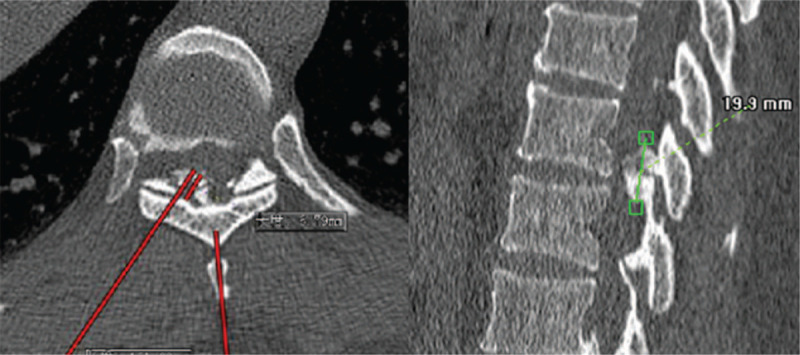
Thickness and height measurement of the ossification on the preoperative CT scans.

**Figure 2:**
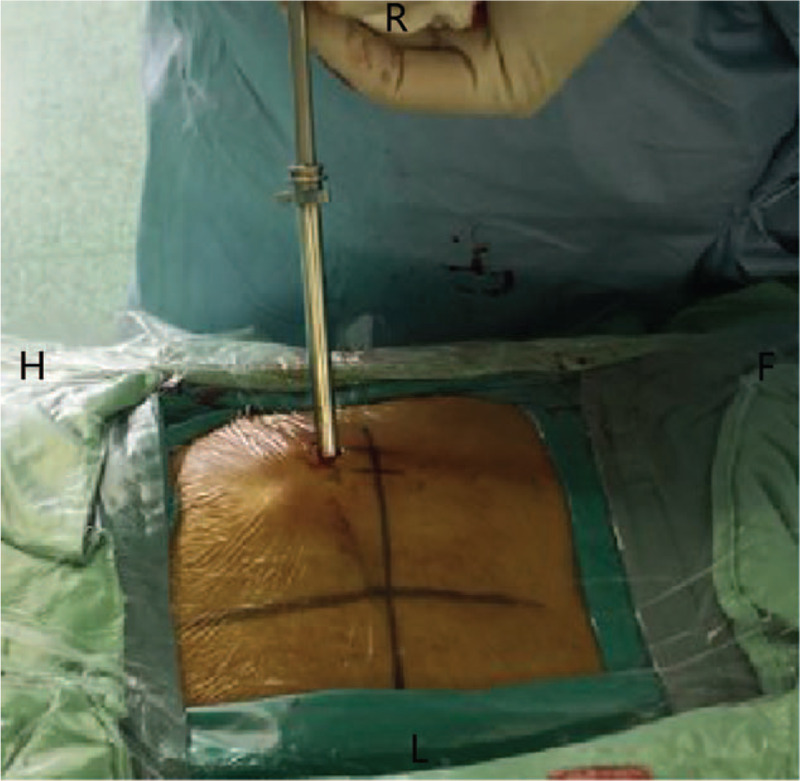
The planed entry point and puncture path on the skin surface.

**Figure 3:**
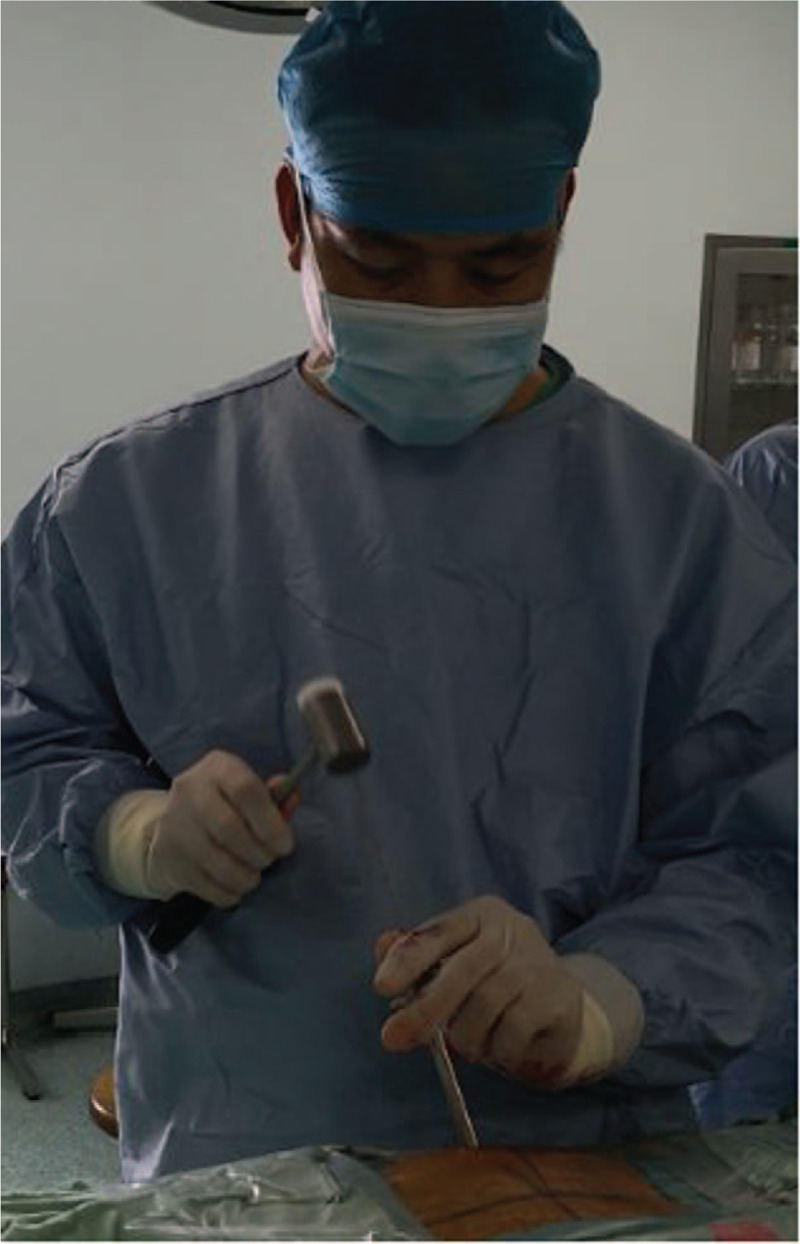
The Kirschner wire nailed to the lamina. F = caudal, H = cephalad, L = left, R = right.

**Figure 4:**
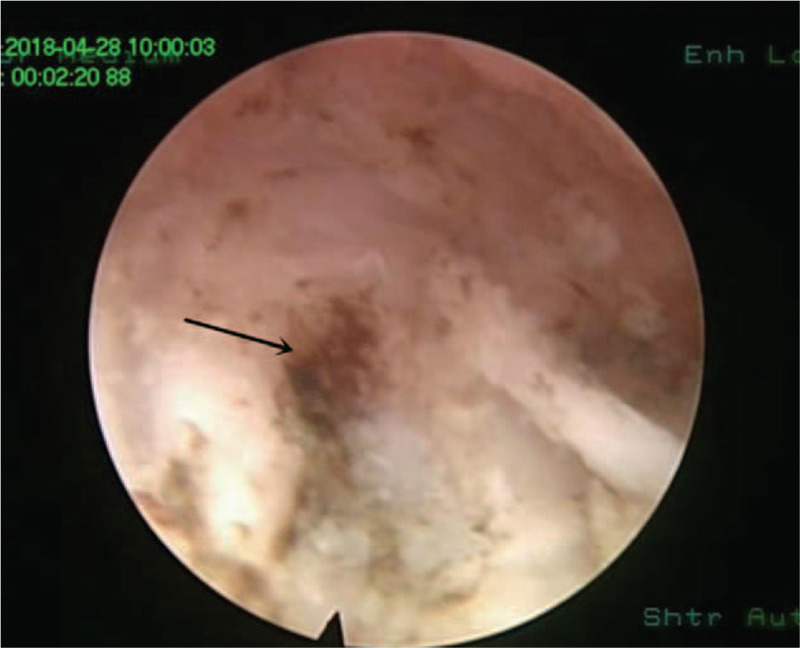
The “bone pit” left on the lamina surface as the arrow showed.

**Figure 5:**
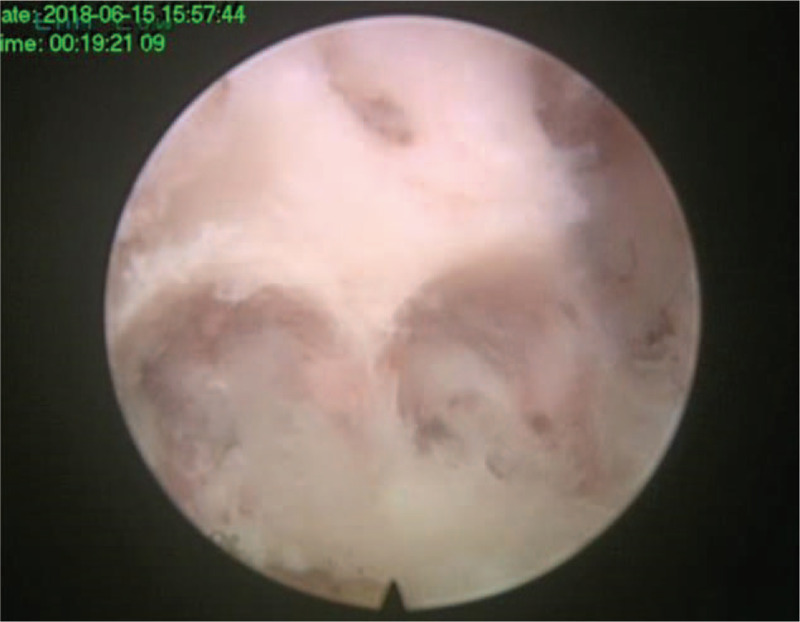
A 3.2mm-diameter drill was used as a scale to estimate the size of lamina opening.

**Figure 6:**
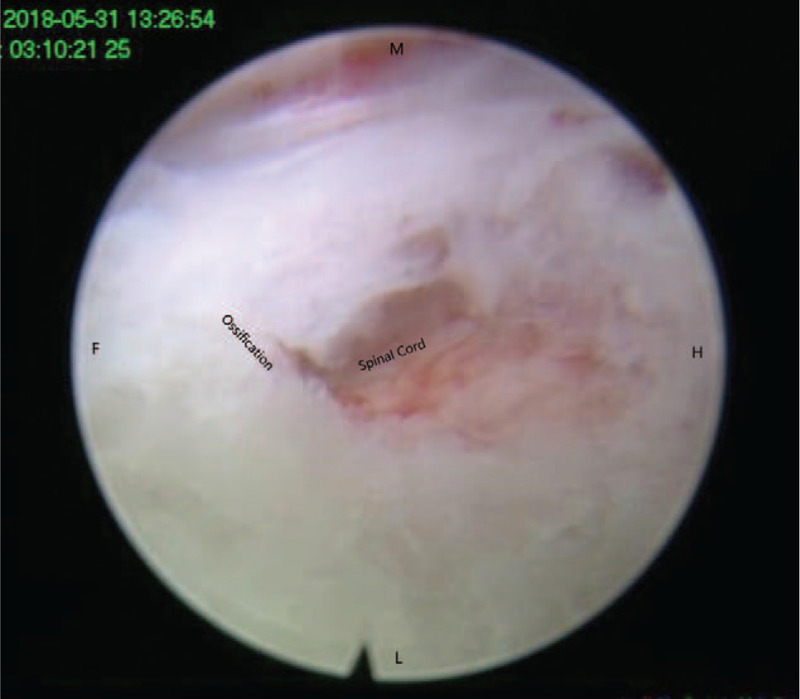
Breakage of the spinal canal from suitable space between the ossification and spinal cord. F = caudal, H = cephalad, L = lateral, M = median.

**Figure 7:**
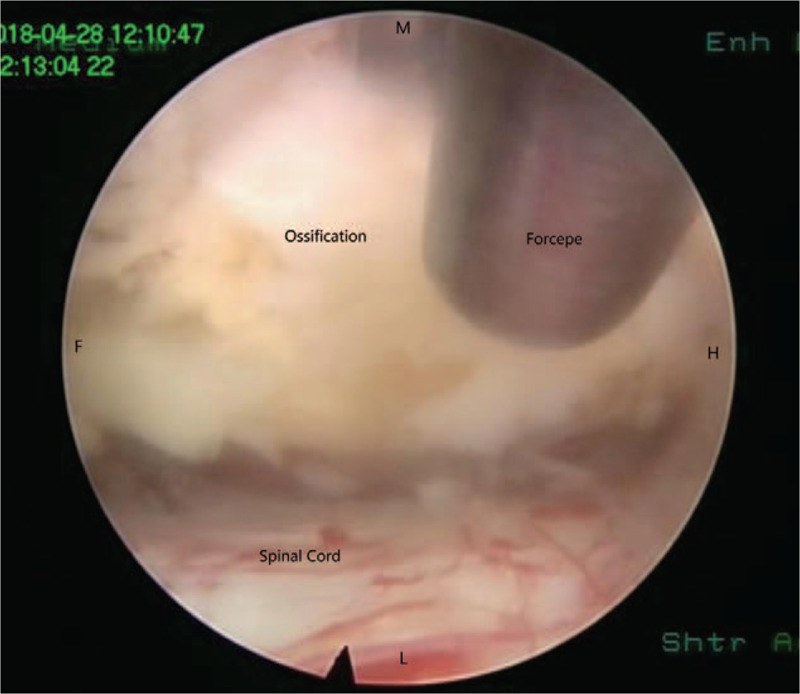
Ossification removed by the forcep. F = caudal, H = cephalad, L = lateral, M = median.

**Figure 8:**
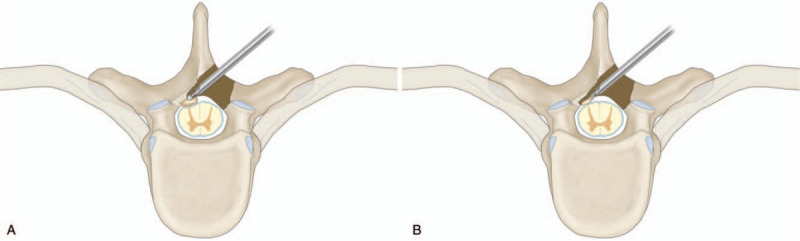
A diagram demonstrating the “over the top” technique. A Ossification detached from the base. B Ossification removed by the forcep.

**Figure 9:**
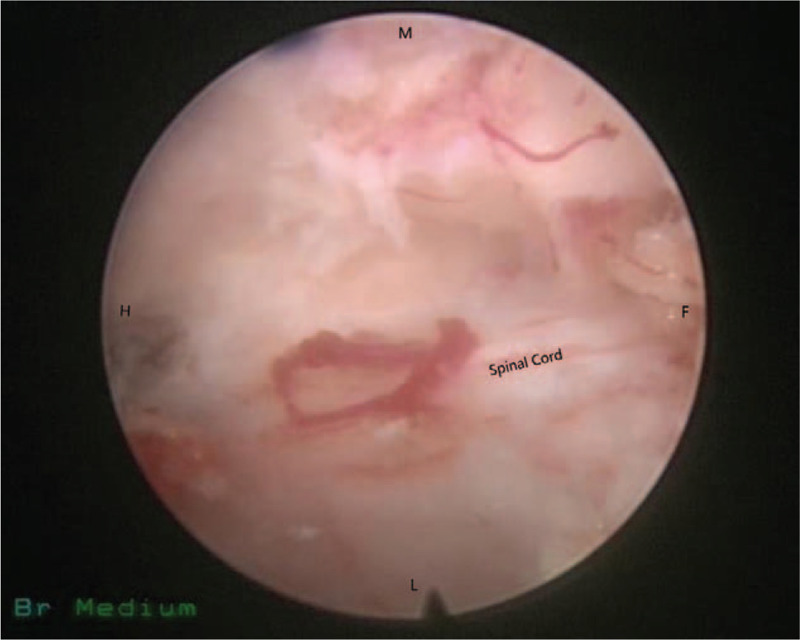
The spinal cord after decompression. F = caudal, H = cephalad, L = lateral, M = median.

According to the clinical manifestations, preoperative CT scans, sagittal reconstructions and magnetic resonance imaging (MRI) results, the approach side was determined. In general, the approach side was chosen as the side with severe imaging compression and severe clinical symptoms. The thickness of the vertebral lamina and the thickness and height of OLF were measured from the imaging data before the operation to assess the extent of decompression (Fig. 1). The patient lied prone on the operating bed with an iliac cushion. The anesthesia method used was local anesthesia with oxygen inhalation and monitoring of ECG, blood pressure, and oxygen saturation. Dexmedetomidine (4 μg/mL) was pumped at a rate of 3 to 8 mL/h to alleviate pain and anxiety. Local anesthetics included a mixture of 0.5% lidocaine and 0.25% ropivacaine. The target segment and puncture path were determined under the guidance of C-arm fluoroscopy. The puncture angle was typically 10 to 15 degrees of head inclination, and the skin entry point was 60 to 80 mm away from the midline (Fig. 2). After adequate soft tissue and lamina surface anesthesia, the Kirschner wire was first punctured into the surface of the lamina. After being adjusted to the center of the operation zone, the Kirschner wire was nailed into the lamina (Fig. 3). The dilator, operating sheath, and endoscope were introduced subsequently along the Kirschner wire. The remainder of the procedure was performed with full endoscopy. The maximum height of the irrigation fluid bag was 1.5 meters above the operation level. After the soft tissue on the surface of the lamina was cleared, the Kirschner wire anchored to the lamina was pulled out under the visual field. Because there was no obvious anatomical marker in the thoracic intervertebral space, the “bone pit” left on the surface by the Kirschner wire could be used as a reference marker for laminectomy (Fig. 4). Breakage of the spinal canal began at a suitable space between the ossification and spinal cord. The grinding drill was an important tool used in this procedure. It was not only used for bone resection but also as a scale (diameter of the drill) to estimate the range of decompression (Fig. 5). Breakage of the spinal canal began at the cranial, cauda, or side of ossification because there was a suitable safe space between the ossification and spinal cord (Fig. 6). The touch of the drill could bring serious consequences to the spinal cord. After thinned, the ossification was separated with the spinal cord by the probe and removed by the forceps or Kerrison rongerurs (Fig. 7). Dorsal and contralateral decompression with the use of the “over the top” technique started after ipsilateral spinal cord decompression was completed.^[4]^ After removal of the base of the spinous process, the drill could reach the contralateral ossification and ventral side of the lamina. After the base detouched from the lamina, Ossification could be removed by forceps (Fig. 8). To some extent, difficulty of the contralateral operation was related to the distance between the puncture entry point and the midline. Proper distance increase was beneficial to the operation. For DO, the ossification was thinned and reduced with a diamond burr, and then it was removed with forceps. Due to the good soft tissue coverage, no interventions were needed for dural tears. Free-floating dura mater in the irrigation fluid at the proximal, distal, ipsilateral, and contralateral sides of the operative field was a sign of sufficient decompression (Fig. 9). The basic operating steps can be summarized as thining-separating-removing. The operative field was perfused with fluid sufficiently before removal, and the endoscope was withdrawn slowly to avoid dural hernia (see Supplemental Video, which demonstrates the endoscopic decompression procedure for thoracic ossification of ligamentum flavum).

On the first day after the operation, the patient was allowed to get out of bed under the protection of braces after magnetic resonance and CT scans. Vigorous exercise was avoided for 6 weeks after the operation. Nonsteroidal anti-inflammatory drugs were administered orally for 1 week, and neurotrophic drugs were administered orally for 1 to 3 months according to the patient's symptoms.

All procedures were performed by the samesurgeon. The data were routinely recorded pre- and postoperatively and after 1 day, 12 weeks, 6, 12 and 18 months. MRI results were obtained at the follow-up times of 1 day, 12 weeks, 6 and 18 months. All patients were followed for 6 to 20 months. The patients were assessed in person during the follow-up. Pre- and postoperative neurological statuses were evaluated using the modified Japanese Orthopedic Association (mJOA) score.^[8]^ The recovery rate (RR) was calculated as follows: RR = (postoperative mJOA-preoperative mJOA)/(11-preoperative mJOA) × 100%. According to the RR, the surgical results were divided into good (50%–100%), fair (25%–49%), unchanged (0%–24%), or deteriorated (< 0%).^[9]^ The visual analogue scale for thoracic back pain was also applied to evaluate thoracic pathologies.

## 4 Results

This study included 6 male patients and 8 female patients aged 36 to 67 years with an average of 56.71 ± 9.15 years and an average follow-up of 19.6 months (range 8–22 months). Two lesions were located in the upper thoracic spine (T1–T4), 11 in the lower thoracic vertebrae (T9–T12) and 1 at the thoracolumbar junction (T12–L1). Patients’ symptoms included numbness and weakness in the lower extremities as well as gait instability and dysfunction of the bladder and sphincter. Back pain was observed in some patients but not all. The duration of preoperative symptoms ranged from 2 to 60 months. Ten patients also had cervical spinal stenosis, lumbar spinal stenosis and/or thoracic spinal stenosis at other segments. However, all the combining factors were discussed and identified as non-major causes according to the clinical and imaging manifestations. Increased signal intensity (ISI) on T2-weighted magnetic imaging was observed in 4 patients (Table 1).

**Table 1:**
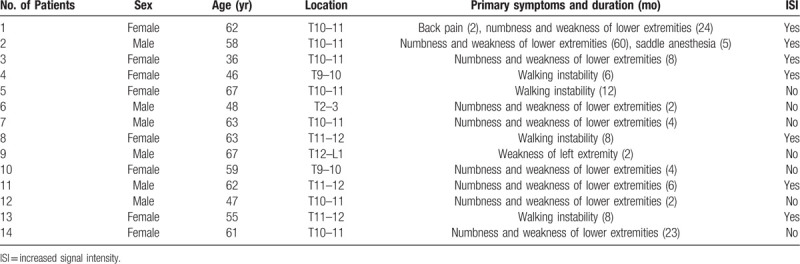
Clinical and imaging characteristics.

The average operation time was 159.73 ± 62.09 minutes, and the hospital stay was 7.43 ± 1.79 days (Table 2). The CT and MRI results on the first day after the operation confirmed that the ossification was completely resected and that spinal cord decompression was sufficient. All patients presented here showed relief or termination of their symptoms. Significant differences were found between preoperative mJOA and mJOA scores at the last follow-up (*P* < .001). Five patients had dural tears during the operation, of whom 4 also had DO. However, no leakage of cerebrospinal fluid from the wound or imaging pseudo-dural cysts occurred after the operation. One patient presented with intraoperative neurological deficits and was given 1000 mg methylprednisolone intravenously, which recovered 30 minutes later. Headache and neck pain occurred during the operation in 1 patient with a dural tear. After lowering the height of irrigation and speeding up the dexmedetomidine pump, the symptoms were alleviated, and the patient achieved completion of the operation, which persisted after surgery. No abnormalities were found according to the head and cervical MRI results. Symptoms disappeared 1 week later (Table 2, Table 3).

**Table 2:**
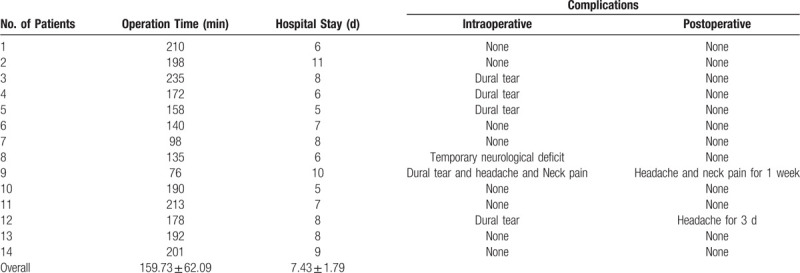
Operation time, length of hospital stay, and complications.

**Table 3:**
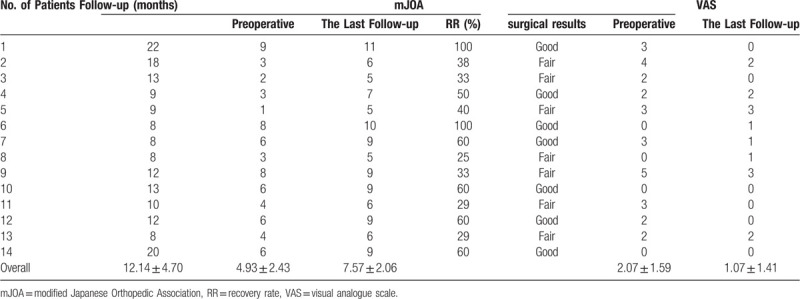
Clinical effect evaluation.

### 4.1 Examples of cases

**Figure 10:**
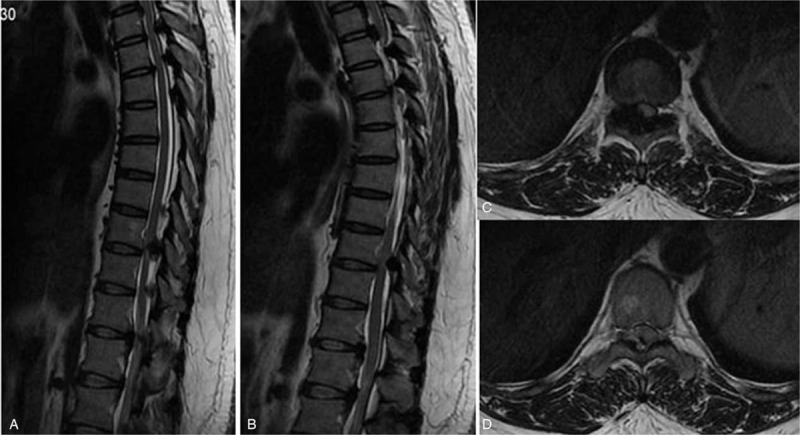
Preoperative magnetic resonance imaging: T9 and 10 ossification of the ligamentum flavum compression of the spinal cord.

**Figure 11:**
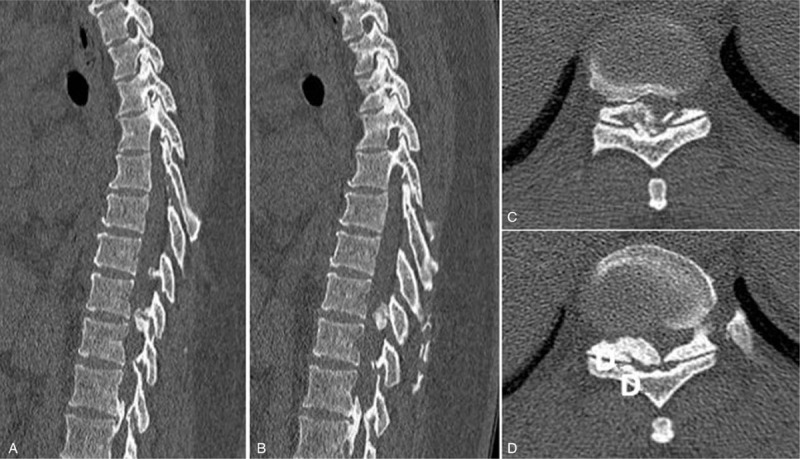
Preoperative CT: T9 and 10 ossification of the ligamentum flavum with dural ossification.

**Figure 12:**
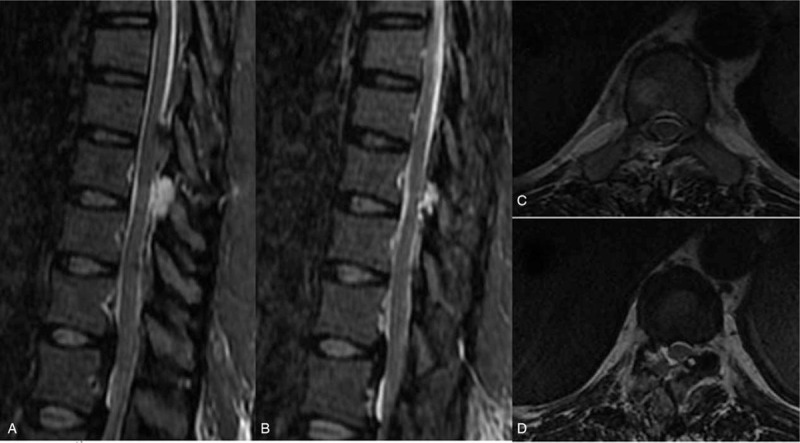
Magnetic resonance imaging on the first day after surgery: decompression of the T9 to 10 spinal canal was sufficient.

**Figure 13:**
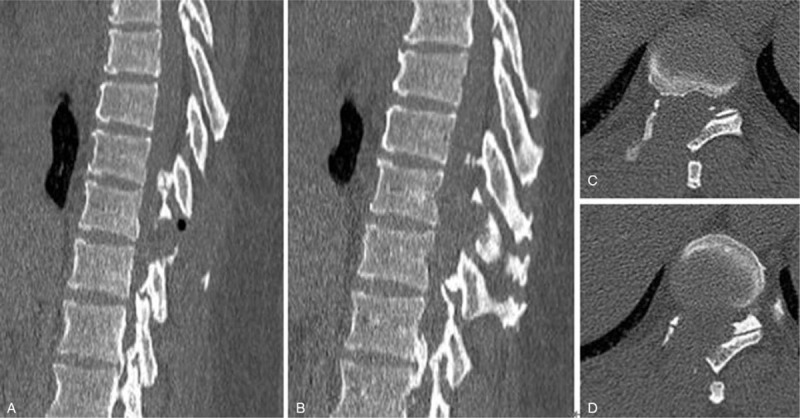
CT on the first day after surgery: Complete resection of ossification of the ligamentum flavum and dural ossification.

Case 1: A female, 46 years old, complained of unstable walking for 26 months and had an mJOA score of 4 points. The pathogenic factor was OLF combined with DO at the T9 and 10 segment (Figs. 10 and 11). Postoperative MRI and CT confirmed that decompression was sufficient (Figs. 12 and 13).

**Figure 14:**
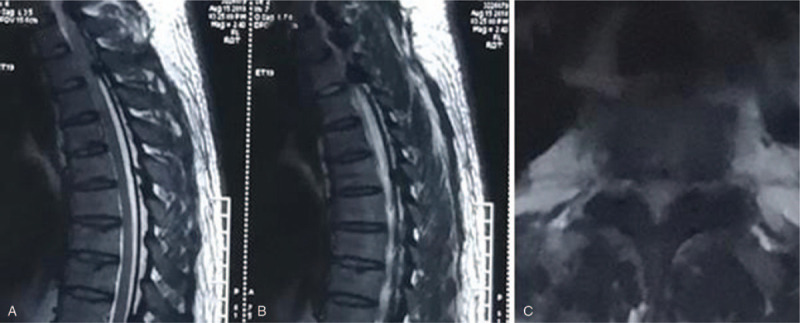
Preoperative magnetic resonance imaging: T2 and 3 ossification of the ligamentum flavum compression of the spinal cord.

**Figure 15:**
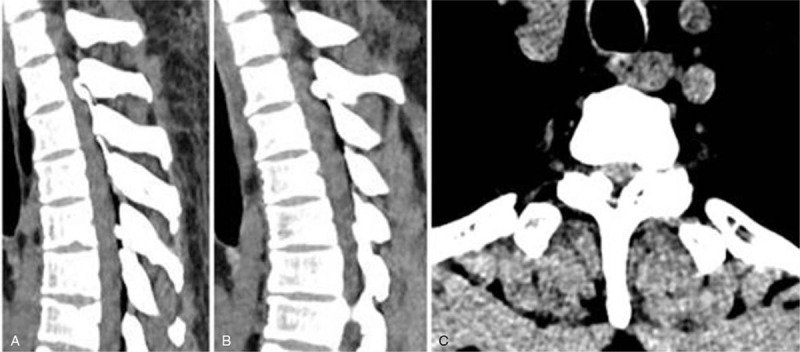
Preoperative CT: T2 and 3 ossification of the ligamentum flavum compression of the spinal cord.

**Figure 16:**
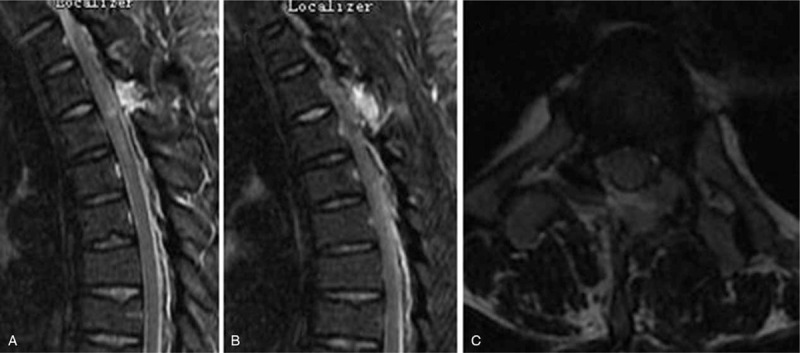
Magnetic resonance imaging on the first day after surgery: decompression of the T2 to 3 spinal canal was sufficient.

**Figure 17:**
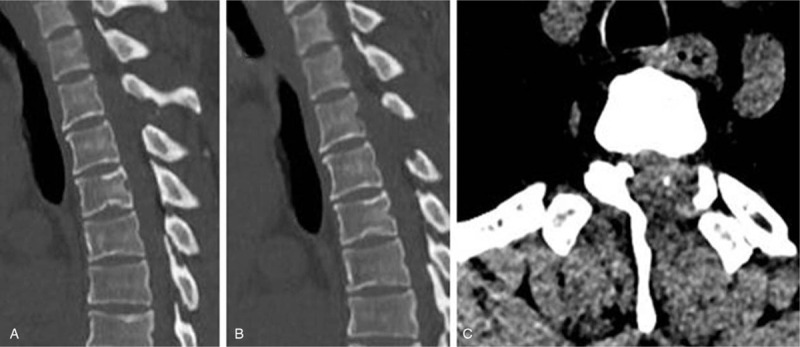
CT on the first day after surgery: sufficient resection of the T2 and 3 ossification of the ligamentum flavum.

Case 2: A male, 48 years old, complained of numbness and weakness of the lower extremities for 2 months and had an mJOA score of 8 points. The pathogenic factor was OLF at the T2 and 3 segment (Figs. 14 and 15). Postoperative MRI and CT confirmed that decompression was sufficient (Figs. 16 and 17).

## 5 Discussion and conclusions

We found that full-endoscopic decompression for thoracicossification of the ligamentum flavum was technically feasible. Sufficient decompression of the spinal cord was observed under endoscopic view as well as in postoperative images. Short-term clinical outcomes were also satisfactory. No serious complications, such as post-thoracotomy pain syndrome, impaired wound healing, infections, or thrombosis, were found.^[10]^ However, the long operation time was an obvious disadvantage.

Good clinical outcomes of full-endoscopic decompression surgery via the interlaminar approach for lumbar stenosis have been reported.^[11]^ We translated this technique to the thoracic spine. There are still some differences between the 2 technologies. Unlike the goal of decompression of the hypertrophic ligamentum flavum in lumbar surgery, the goal of thoracic spinal canal decompression is ossification. Exposure of the thoracic spinal cord is mainly accomplished with the aid of drilling. The spinal cord cannot be compressed during the whole operation. The risk and difficulty are very high. Some of the skills required for the operation are as follows:

1. because there is no obvious anatomical marker on the surface of the thoracic lamina, the Kirschner wire anchored to the lamina can confirm the operative zone;
2. spinal cord exposure should begin in a space between the ossification and the spinal cord, and the exposure of the spinal cord can be initiated from the cranial, caudal or midline space, which is determined according to the preoperative imaging analysis; and
3. the distance between the incision and the midline depends on the size of the contralateral ossification and its degree of compression on the spinal cord. Generally, 6 to 8 cm is suitable. In our study, 1 patient with OLF in the T12-L1 segment was confirmed to have undergone complete removal of the contralateral ossification by endoscopy. However, dural tears occurred during this process. The bundles floating in the operation area affected decompression in the contralateral ventral part of the spinal cord.

In our study, the average improvement in the mJOA score was 2.64 in 14 patients at the last follow-up. Osman NS et al reviewed 137 patients with thoracic OLF who underwent total laminectomy. The mean improvement in the mJOA score was 3.03.^[12]^ Zhang J et al suggested that the preoperative ISI and duration of symptoms were risk factors for surgical outcome in patients with thoracic OLF. In particular, a preoperative signal change ratio ≥1.54 and symptom duration >12 months were significant risk factors for a poor surgical outcome.^[13]^ In this study, 7 out of 14 patients had ISI. The mean preoperative symptom duration was approximately 7 months. Perhaps the 2 factors affected the outcome of surgery. Moreover, the recovery time was short, and the improvement in nerve function needs further observation.

Dural tears represent the most common complication of open laminectomy surgery. The incidence is 18.4%. The incidence of dural tears with clinical symptoms is 12.1%.^[12]^ The occurrence of dural tears is highly correlated with DO.^[4,14]^ In this study, DO was confirmed in the endoscopic view in 4 of 5 patients with dural tears. This was why the incidence of dural tears was higher than in previous reports. An et al performed similar endoscopic decompression surgery for the treatment of thoracic OLF in 18 patients. The incidence of dural tears was 11.1%.^[15]^ However, patients with DO were excluded from their study.

Although a dural tear was found during the operation, no intervention was needed, such as suturing or covering. None of the 5 patients had poor healing of the incision or a clinical or imaging pseudo-dural cyst. This may be because the incision and soft tissue damage were very light due to the use of minimally invasive surgery. Ruetten S, Xiaobing Z and others reported that no dural defects were repaired during endoscopy surgery and that no complications, such as cerebrospinal fluid leakage, occurred after the operation.^[10,16,17]^ Dural tears seem not to be a major complication in full-endoscopic spinal surgery, but this result still needs further confirmation, and whether dural tears are related to the decompression range, the diameter of the working sheath or the distance of soft tissue travel is not clear.

Intraoperative and postoperative headache and neck pain are special complications of endoscopic spinal surgery. The reason is related to the increase in cerebrospinal fluid pressure under continuous water irrigation. When the epidural pressure is greater than 37 mm Hg, the incidence of cervical pain increases significantly.^[18]^ Another complication of thoracic spinal decompression is transient or persistent neurologic deficits. The incidence in open surgery under general anesthesia is 5% to 15%. The possible reasons are as follows:

1. injury of the spinal cord blood supply;
2. direct injury during the operation; and
3. postoperative hematoma.^[5,19]^

The application of neuroelectrophysiological monitoring during open thoracic spinal decompression greatly reduces the probability of postoperative neurological deficits. However, there are still some false-positives or false-negative results. In the few reports on the use of full-endoscopic surgery for thoracic OLF under local anesthesia, there have been no reports of continuous neurologic deficits.^[10,16,17]^ Full-endoscopic thoracic decompression is usually performed under local anesthesia, and patients can promptly provide feedback during the stimulation of the spinal cord. This procedure is safer than open surgery under general anesthesia. However, because full-endoscopic thoracic surgery has higher technical requirements for the surgeon and the operation time is longer, any accidental injury to the spinal cord may lead to serious consequences.

The long operation time is a disadvantage of this surgery. With the improvement of endoscopic instruments, the efficiency of surgery may be further improved. The hospital stay was longer in our study because we needed a long period of clinical observation to ensure the safety of the surgery. Our study also has certain limitations, including a short follow-up time, the incomplete evaluation of outcomes, and the lack of case-control groups. Most of the follow-up times reported in the literature are more than 18 months.^[1,13,14]^ In particular, the recovery of spinal cord function requires a longer process. Complications such as OLF progression and secondary kyphosis require long-term follow-up.

In conclusion, our results suggest that full-endoscopic decompression for thoracic OLF is achievable. It is superior to open laminectomy surgery in terms of some complications, such as dural tears and neurologic deficits. Our findings indicate that full-endoscopic decompression surgery is a safe and effective choice for the treatment of thoracic OLF even cases with DO.

## Acknowledgments

Thanks are due to Miss Hua Wan for assistance with the writing.

## Author contributions

Wenyi Li: Study Design, Statistical Analysis, and Manuscript Preparation.

Shangju Gao: Data Collection, Literature Search and Manuscript Preparation.

Long Zhang: Data Collection and Statistical Analysis.

Can Cao: Data Collection and Literature Search.

Jingchao Wei: Data Collection and Literature Search.

## Supplementary Material

Supplemental Digital Content

## References

[1] Aizawa T Sato T Sasaki H Thoracic myelopathy caused by ossification of the ligamentum flavum: clinical features and surgical results in the Japanese population. J Neurosurg Spine 2006;5:514–9. 17176015 10.3171/spi.2006.5.6.514

[2] Inamasu J Guiot BH A review of factors predictive of surgical outcome for ossification of the ligamentum flavum of the thoracic spine. J Neurosurg Spine 2006;5:133–9. 16925079 10.3171/spi.2006.5.2.133

[3] Ahn DK Lee S Moon SH Ossification of the ligamentum flavum. Asian Spine J 2014;8:89–96. 24596612 10.4184/asj.2014.8.1.89 PMC3939377

[4] Komp M Hahn P Oezdemir S Bilateral spinal decompression of lumbar central stenosis with the full-endoscopic interlaminar versus microsurgical laminotomy technique: a prospective, randomized, controlled study. Pain Physician 2015;18:61–70. 25675060

[5] Komp M Hahn P Ozdemir S Operation of lumbar zygoapophyseal joint cysts using a full-endoscopic interlaminar and transforaminal approach: prospective 2-year results of 74 patients. Surg Innov 2014;21:605–14. 24667524 10.1177/1553350614525668

[6] Palumbo MA Hilibrand AS Hart RA Surgical treatment of thoracic spinal stenosis: a 2- to 9-year follow-up. Spine (Phila Pa 1976) 2001;26:558–66. 11242384 10.1097/00007632-200103010-00021

[7] Xiaobing Z Xingchen L Honggang Z U” route transforaminal percutaneous endoscopic thoracic discectomy as a new treatment for thoracic spinal stenosis. Int Orthop 2019;43:825–32. 30218183 10.1007/s00264-018-4145-y

[8] Zhang J Wang L Li J Predictors of surgical outcome in thoracic ossification of the ligamentum flavum: focusing on the quantitative signal intensity. Sci Rep 2016;V6N:23019. 10.1038/srep23019 PMC4785339 26960572

[9] Yonenobu K Ebara S Fujiwara K Thoracic myelopathy secondary to ossification of the spinal ligament. J Neurosurg 1987;66:511–8. 3104552 10.3171/jns.1987.66.4.0511

[10] Chang UK Choe WJ Chung CK Surgical treatment for thoracic spinal stenosis. Spinal Cord 2001;39:362–9. 11464309 10.1038/sj.sc.3101174

[11] Osman NS Cheung ZB Hussain AK Outcomes and complications following laminectomy alone for thoracic myelopathy due to ossified ligamentum flavum: a systematic review and meta-analysis. Spine (Phila Pa 1976) 2018;43:E842–8. 29940604 10.1097/BRS.0000000000002563 PMC6252088

[12] Ruetten S Komp M Merk H Full-endoscopic interlaminar and transforaminal lumbar discectomy versus conventional microsurgical technique: a prospective, randomized, controlled study. Spine (Phila Pa 1976) 2008;33:931–9. 18427312 10.1097/BRS.0b013e31816c8af7

[13] Quillo-Olvera J Lin GX Kim JS Percutaneous endoscopic cervical discectomy: a technical review. Ann Transl Med 2018;6:100–13. 29707549 10.21037/atm.2018.02.09 PMC5900065

[14] Hou X Chen Z Sun C A systematic review of complications in thoracic spine surgery for ossification of ligamentum flavum. Spinal Cord 2018;56:301–7. 29284792 10.1038/s41393-017-0040-4

[15] An B Li XC Zhou CP Percutaneous full endoscopic posterior decompression of thoracic myelopathy caused by ossification of the ligamentum flavum. Eur Spine J 2019;28:492–501. 30656471 10.1007/s00586-018-05866-2

[16] Hur H Lee JK Lee JH Thoracic myelopathy caused by ossification of the ligamentum flavum. J Korean Neurosurg Soc 2009;46:189–94. 19844616 10.3340/jkns.2009.46.3.189 PMC2764014

[17] Ruetten S Hahn P Oezdemir S Full-endoscopic uniportal decompression in disc herniations and stenosis of the thoracic spine using the interlaminar, extraforaminal, or transthoracic retropleural approach. J Neurosurg Spine 2018;29:157–68. 29856303 10.3171/2017.12.SPINE171096

[18] Joh JY Choi G Kong BJ Comparative study of neck pain in relation to increase of cervical epidural pressure during percutaneous endoscopic lumbar discectomy. Spine (Phila Pa 1976) 2009;34:2033–8. 19675511 10.1097/BRS.0b013e3181b20250

[19] Li Z Ren D Zhao Y Clinical characteristics and surgical outcome of thoracic myelopathy caused by ossification of the ligamentum flavum: a retrospective analysis of 85 cases. Spinal Cord 2016;54:188–96. 26238315 10.1038/sc.2015.139

